# Anæsthetics in Dental Practice

**Published:** 1891-03

**Authors:** J. Cowan Woodburn

**Affiliations:** Lecturer in Glasgow Dental Hospital


### ANÆSTHETICS IN DENTAL PRACTICE.[1]

BY J. COWAN WOODBURN, M.D., LECTURER IN GLASGOW DENTAL HOSPITAL.

Owing to the limited time apportioned to the speakers on this problem,—the science and art of chloroform anæsthesia,—I am forced to content myself with a few thoughts on the subject from the purely practical and ethical aspect. These being only culled from my original design, consequently will be few and probably of a rambling character. First, then, the selection of an anæsthetic is a question of great importance. I do not touch upon the personal points which render patients suitable for the anæsthetic condition, but may state that I do not remember ever having denied this boon when I deemed the severity of the operation rendered it justifiable; at the same time denying it when I conscientiously considered that by its administration I was simply pandering to an ungrounded and silly fear. I should consider myself sadly wanting in professional resources, in such cases, if I could devise no other means of allaying unfounded trepidation than by the complete overthrow of reason and the obliteration of sense and feeling. On the other hand, I am constrained to maintain that, even in cases of organic disease, the grounds on which the administration of an anæsthetic are denied are of a very slender and doubtful character; and even when a severe operation has to be undergone, in the presence of organic disease, I am distinctly of opinion that the chances of safety are greater *with* the anæsthetic carefully administered, the powerful shock to the system which would otherwise be caused being thus obviated. Again, anæsthetics, we should bear in mind, are successfully administered with great frequency in cases of organic disease, where its existence has never been suspected. Again, in the great bulk of cases of death from anæsthetics post-mortem examination reveals no appreciable lesion whatever, and, in a third class, the existence of organic disease is only disclosed during the process of anæsthesia. Of the latter example, a case recently came under my notice in a young lady of average healthy appearance. No suspicion leading to the use of the stethoscope was excited by objective phenomena. When about to operate, after the production of anæsthesia, a visible regurgitation was noticed in

[1] Read before the Faculty of Physicians and Surgeons of Glasgow, Scotland, October, 1890.

the vessels of the neck. Other things being well, I proceeded with the operation. Having taken the patient, after recovery, into my confidence, she coolly confessed to me that she knew she was laboring under heart-disease, but did not speak of it for fear I should refuse to administer chloroform to her. The *care* which *should* be taken in every administration of chloroform is adequate even in cases of known existing disease.

In what we call large operations in our department of surgery, —that is, when a great number of diseased teeth and roots are to be removed,—in order that the patient may not suffer from the depressing effects of anticipation, and the frequent repetition of operations, it is, as a general rule, best to administer chloroform or ether. Of the latter I have not had much experience, and thus do not consider myself justified in speaking regarding it; but this I may say, I recently performed a large operation under its influence, which was in *every way* highly satisfactory. An especial feature was the speedy recovery, almost equal to that of nitrous oxide gas, in all sufficiently encouraging to recommend its use. If both jaws are to be operated on, it is usual to recommend—unless unusually favorable conditions exist, such as well-absorbed alveoli and little hemorrhage—to divide the operation into two periods, with as long an interval between them as general circumstances will admit.

Medical men in giving chloroform for a dental operation very generally have a dread of the blood, which is at first very copious, getting into the air-passages. In one case which occurs to me, a well-known surgeon of this city, now deceased, insisted on plugging each socket as a tooth or root was extracted. I do not remember in all my experience having seen a case where bleeding in this manner gave trouble save in temporarily concealing the teeth to be removed and thus complicating matters for the operator. My care has been more directed to prevent the chances of its being swallowed, so as to obviate the severity of the sickness and vomiting, having a conviction that one cannot digest one's own blood ; and which, if not wholly ejected from the stomach (in the coffee-grounds condition), produces constitutional irritation, such as fever and malaise, in proportion to the amount retained.

In falling back of the tongue, a not uncommon occurrence, I find especially young practitioners diving at it with artery forceps, heedless of laceration and subsequent obloquy. I do not remember ever having used the forceps, finding it equally efficient to grasp the lower jaw with my left hand and with my forefinger well at the back of the tongue pull the whole forcibly forward.

With regard to position in our operations, it goes without saying that the perfectly recumbent position is against the interests of the dental operator, inasmuch as the angle at which he is to work is peculiarly disadvantageous. I remember in my father's practice, and in my own early experience, sitting in the operating-chair was the customary position, not much removed from that for other dental operations. I do not advocate in favor of incomplete recumbent position, but, for reasons to be afterwards advanced, it would not be culpable to deviate from it to some extent and upon explainable grounds. I would not have you regard me in open rebellion with all acknowledged authority, nor incur the criticism of the Hyderabad Commission, who claim this as "No. 1" in their practical conclusions; but, in fulfilment of my intention when I began, I am bound to relate my own personal experiences.

Nitrous oxide is undoubtedly a valuable anæsthetic to the dentist, and ought to be to the general practitioner. Its unquestionable drawback in lengthy operations is the transitory nature of its effects, an advantage of safety, but one which minimizes its popularity in general surgery. If well administered,—I mean by that the perfect exclusion of atmospheric air,—the anæsthesia is complete and nearly always universal in its physical demonstrations. It is perhaps to this feature that it owes its popularity as a safe agent, it being not so subtle nor deceptive as its ally, chloroform. The operator can determine the changes it is producing in the great nerve-centres more plainly, and coincident with the cutting off of the supply recovery is commenced,—*i.e.*, a steady return to the standard oxygenation of the blood. I am astonished that it is not more frequently used in short and painful operations in general surgery, but, as it is, it is the peculiar property of dental surgeons; and it is the more astonishing, seeing that the popular mind is assured of its complete safety. I have given it frequently to medical friends, for operations such as opening abscesses, cutting carbuncles, and the other night administered it with complete success while a medical friend excised two tonsils and a mass of adenoid growths from the naso-pharynx in a girl of about ten years. In many cases of painful and, if you choose, minor operations in general surgery, if a man is well informed as to what he has to do, and prepared to do it dexterously, he will find this anæsthesia sufficiently deep and prolonged, and save his patient the discomfort of the after-effects of chloroform. I feel constrained to ask surgeons to give it a trial in parallel cases, and my willing services will be at their command. It is but right to state that nitrous oxide has been objected to on the ground of its causing certain mental perversions, but in my experience I have not encountered much of this, as their occurrence, to a large extent, may be anticipated by confident assurance and general comportment on the part of the operator.

I hardly intend to speak of deaths or threatened deaths under gas or chloroform, expecting the discussion of it in abler hands than mine, but I may be excused a remark or two on this head. We all know that death takes place not unfrequently from chloroform, that such incidents are frequently brought under public notice by the lay press, and we know also that not a small percentage of chloroform deaths are never heard of beyond the operating-room, while the same issue so rarely accompanies the administration of nitrous oxide that fatal cases might be numbered on the fingers; the only one that occurs to my memory, other than the Exeter case which happened some years ago, and in which the fatal result was due to the gag getting into the windpipe, is that of a notable lady in Edinburgh; and this case furnishes a good subject for signalizing one or two points worthy of being alluded to. In this case death did not occur under the anæsthetic, but followed directly as the result of it. The patient was depressed by nervous apprehension of the operation, and had expressed her conviction that she would die under it. This depressing influence was brought to bear upon a fatty heart, and determined the fatal syncope. Two other conditions to which I beg your attention: The dress fitted so tightly that its removal was accomplished with difficulty, and the stomach was distended with undigested food, part of which was vomited during the performance of artificial respiration. The *Lancet*, in reviewing all the circumstances, said: "There is no reason for going back from the position which modern anæsthetists have occupied, that nitrous oxide is not only the safest anæsthetic we possess, but that when it is circumspectly given, it is practically free from danger save in cases of very advanced diseases of the heart or lungs." The *British Medical Journal*, after a dispassionate consideration, blamed the neglect of precautions on the part of the patient rather than to any particular danger from the anæsthetic. Now, from the fact that this lady had taken food early in the morning and that she was not operated upon till noon, and that even then the stomach contained undigested food, we are reminded of the physiological truth that during restraint of heavy nervous apprehension the functions of digestion are largely arrested, and hence we should be guided in our recommendations modifying our dietary instructions in accordance with the physiological requirements of the case. In pregnency I had at first some misgiving as to the administration of gas, but in this condition I have frequently administered it without any outward symptoms except a tendency to vomit, which is not common in ordinary circumstances. In valvular disease of the heart there is a tendency to syncope with gas. Among other phenomena may be noticed increase of the pulse, increased cardiac force, loss of tidal wave, accentuation of dicrotic curve, and opisthotonos most common in females. The pupils are generally dilated, but this is not to be depended upon as a test of the narcosis. It is worth remarking, on ethical grounds, that erotic movements and sexual illusions are not uncommon.

Just a word about idiosyncrasy in chloroform anæsthesia. This is a point that I have merely alluded to, and I feel it is of more importance than is generally thought of, at least sufficient to justify its being looked upon as an item of *attentio* in administering the drug to a patient for the first time. As to how it acts differently on different individuals would be as easily answered as the well-known idiosyncrasy attending the effects of mercury and some other medicines. Some years ago, before the supremacy of nitrous oxide gas, I had to administer chloroform to a young lady for the removal of a tooth. I used a handkerchief in the form of a bird's nest, and the position of the patient was not quite semi-recumbent, less than a teaspoonful of chloroform was sprinkled, and plenty of atmosphere allowed. She had a large protruding glaucomatous eye which from the first focussed itself upon me with so fixed a glare that in less than half a minute I was induced to put my finger on it. Finding it quite insensate, I removed the napkin and, she being completely insensible, hurriedly extracted the tooth; being still unconscious of the operation, I looked for more bad teeth and had them out. Almost immediately afterwards she awoke entirely well and unconscious of what I had done, and grateful for what I had taken upon myself to do. In another case that I had with the late Dr. J. G. Wilson, in which some ounces of chloroform (I am afraid to say how many) were used to remove a number of bad teeth and an epuloid growth, the lady seemed proof against its influence, and I had to operate while she was still conscious and sensation only slightly obtunded. I mention these two extreme cases to show, on the one hand, a hypersusceptibility, and, on the other, an obstinate tolerance, to justify the consideration of idiosyncrasy.

Let me say a word on the ethical aspect of the subject. I need not say that the after-effects of chloroform are often of a very humiliating character from the nausea and vomiting, and especially so in dental operations, when the blood enters into the stomach; this, combined with the physical prostration and general demoralization, renders it, as a rule, better to have this performed under the influence of home comforts. I never have a chloroform case in my house but my heart goes out to the patient, when I see them hustled into a cab, and I am inclined to crush my thoughts of the misery that is being endured while they rattle over the stony street. This is the social consideration; but still worse remains behind when I think of it from the scientific point of view. I need not say that the patient is thus placed in a position far removed from comfort if not safe recovery. It is also morally better for the patient, to be saved the depressing influence of fear on being brought face to face with the horrors of the surgical armory, when any such may be ameliorated by the kindly influence of domestic surroundings. Time prevents me from detailing the advantages that show, as a rule, that it is preferable to chloroform in the patient's own house. I also cannot enumerate the disadvantages it is to the dental operator, but I am persuaded that these would be outweighed by the paramount consideration,—the welfare of the patient.

The other point which might be considered in this discussion is, In how far is one medical man justified in administering chloroform without the presence and assistance of another? I am persuaded that many here will estimate that it admits of an element of cowardice to seek the help of another, while, in reality, it is the demonstration of real bravery, and an evidence of high moral culture. That which makes up the self-possession of youth is often but the offspring of ignorance or, if you will, inexperience,—and the success of their actions is gained in the game of hap-hazard, but the matured, who have gained experience in the trying ordeals of professional life, can discern more clearly the sands and shoals of the social and professional existence. It is in such, though cloaked from the outer world, that the secret misgivings are strongest. The more his experience teaches him the better he realizes the great mystery, until the culminating end reveals his own littleness and complete dependence. So I am prepared to think that it would be the younger men, and the least experienced, who might dissent from recommending the necessity of a plurality of attendants at the giving of an anæsthetic. It is only those who have grasped the grave responsibility of hanging by a thread the life of a fellow-creature who would not deem it an act of compromise to seek help in bearing the strain; and I am persuaded that there is not a head grown gray in medical service but would approve and urge the advisability of two being present in the production of chloroform anæsthesia, and even encourage legislation in its favor.

Lastly, there are some, again, who would urge against it, on the ground that patients would grudge the expense possibly entailed by the multiplication of medical attendants,—let me say that I never heard it objected to on that ground, even by those who appeared well able to afford it. Be that as it may, to us, members of an acknowledged unselfish profession, other means may be found so that objection may not stand in the way of offering the best human security for a life in imminent peril.

